# An Outbreak of Neuroangiostrongyliasis (Rat Lungworm Disease) in Hawai‘i: Evidence Supporting the Early Use of Albendazole–Corticosteroid Co-Therapy

**DOI:** 10.4269/ajtmh.25-0689

**Published:** 2026-03-31

**Authors:** Vernon Ansdell, John Jacob, William L. Gosnell, Jourdan K. P. McMillan, Johnnie Yates, Louis Lteif, Deniz Bicakci, Pakieli H. Kaufusi, Kenton Kramer

**Affiliations:** ^1^ Department of Tropical Medicine, Medical Microbiology and Pharmacology, John A. Burns School of Medicine, University of Hawai‘i at Mānoa, Honolulu, Hawai‘i;; ^2^ Daniel K. Inouye College of Pharmacy, University of Hawai‘i at Hilo, Hilo, Hawai‘i;; ^3^ Hawai‘i Department of Health, Hazard Evaluation and Emergency Response Office, Pearl City, Hawai‘i;; ^4^ Hawai‘i Permanente Medical Group, Honolulu, Hawai‘i

## Abstract

Neuroangiostrongyliasis (NAS) is an emerging parasitic infection caused by the neurotropic nematode *Angiostrongylus cantonensis* (*A. cantonensis*). It is the leading infectious etiology of eosinophilic meningitis worldwide. In 2017, six men living on Hawai‘i Island developed NAS after drinking homemade kava. After the event, a dead slug, likely infected with *A. cantonensis*, was found at the bottom of a communal vessel. Neuroangiostrongyliasis was subsequently confirmed in three patients through polymerase chain reaction testing of their cerebrospinal fluid. Two patients were presumptively diagnosed with NAS on the basis of highly suggestive signs and symptoms, the presence of eosinophilic meningitis, and a shared history of exposure. A lumbar puncture was unsuccessful in one patient, who was presumptively diagnosed with NAS on the basis of his symptoms and shared exposure history. Five of six patients received at least 2 weeks of co-therapy with albendazole and corticosteroids, which was initiated within 15 days of infection. The treatment was well-tolerated, and no significant adverse events were noted. One patient who did not have health insurance completed only two doses of albendazole because of the cost of the drug and experienced symptom recurrence on several occasions. This rare, common-source outbreak of *A. cantonensis* infection supports the early use of albendazole–corticosteroid co-therapy in proven or suspected cases of NAS. It also provides additional evidence for the water transmissibility of this parasite.

## INTRODUCTION

Neuroangiostrongyliasis (NAS), also known as rat lungworm disease, is caused by the neurotropic nematode *Angiostrongylus cantonensis* (*A. cantonensis*). The disease was first recognized in a Taiwanese patient with eosinophilic meningitis (EOM) in 1945.^1^^,^^2^ Since then, the parasite has been documented in many tropical and semi-tropical parts of Asia, the Pacific, the Caribbean, South America, Australia, the southern United States, and several African countries.^3^ Recent evidence has revealed that the parasite has spread to Europe, including the Canary and Balearic Islands, where infections have been detected in animals such as hedgehogs and lemurs.^4^^,^^5^ Most recently, infection has been described in mammals and mollusks in Spain and Italy;^6^^–^^10^ thus, it is likely that human NAS will eventually be identified in continental Europe. Infections in non-human primates in zoos and private collections, and even in dogs, may act as important sentinels.^8^

Rats (genus *Rattus*) are the definitive hosts of *A. cantonensis*, with numerous gastropod species serving as intermediate hosts.^11^ Human infection typically occurs through the ingestion of raw or undercooked gastropods (slugs and terrestrial and freshwater snails) that are infected with third-stage (L3) larvae of *A. cantonensis*, as well as through the consumption of various paratenic or transport hosts.^11^^,^^12^ The signs and symptoms of NAS likely reflect the number and location of *A. cantonensis* larvae in the central nervous system (CNS) and range in severity from transient paresthesia and acute headaches to significant neurologic syndromes with long-term disability and other sequelae, including paralysis, coma, and even death.^12^^–^^14^

**Figure 1.**
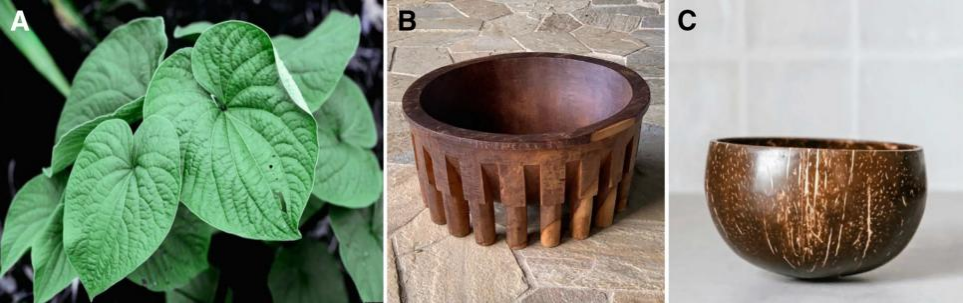
Leaves of a kava plant (**A**), a large serving bowl, 18 inches in diameter (**B**), and a representative cup, 5 inches in diameter, for drinking kava (**C**). Photo credit: Vernon Ansdell, MD.

Drinking kava has been a traditional custom among Pacific Islanders for more than 3,000 years.^15^ It is prepared from the root of the kava plant (*Piperaceae methysticum*; Figure 1A) by rehydrating dried kava root powder with water. The resulting brown, opaque liquid is periodically transferred into a large serving bowl (Figure 1B). Individual portions are served in cups (Figure 1C).^16^ Although kava is used for ceremonial and recreational purposes, the beverage also plays an important role in traditional medicine across the Pacific. Extracts of kava are purported to possess anxiolytic, analgesic, anesthetic, anticonvulsant, and neuroprotective effects.^17^

In this report, a cluster of cases of NAS is described in six men residing on the southeastern side of Hawaiʻi Island, colloquially known as the Big Island of Hawaiʻi, where NAS has been frequently reported.^18^ The individuals became ill after drinking kava, which was subsequently found to be contaminated with at least one slug presumed to be infected with *A. cantonensis*. The patients’ clinical course and management, as well as the circumstances surrounding the incident, are outlined in the present report.

## MATERIALS AND METHODS

### Diagnosis.

Eosinophilic meningitis is a notable feature of NAS. At the time of this outbreak, EOM was defined as the presence of 10 or more eosinophils per microliter of cerebrospinal fluid (CSF), or eosinophils accounting for more than 10% of the white blood cell (WBC) count when there are at least six total WBCs per microliter of CSF.^19^^,^^20^ The definitive diagnosis of NAS is the detection of *A. cantonensis* larvae or the parasite’s DNA in the patient’s CSF. Cerebrospinal fluid samples that met the criteria for EOM were tested at the Hawaiʻi State Laboratory Division for the presence of *A. cantonensis* DNA using real-time polymerase chain reaction (RTi-PCR) testing.^21^

## RESULTS

### Summary of cases and clinical review.

**Figure 2.**
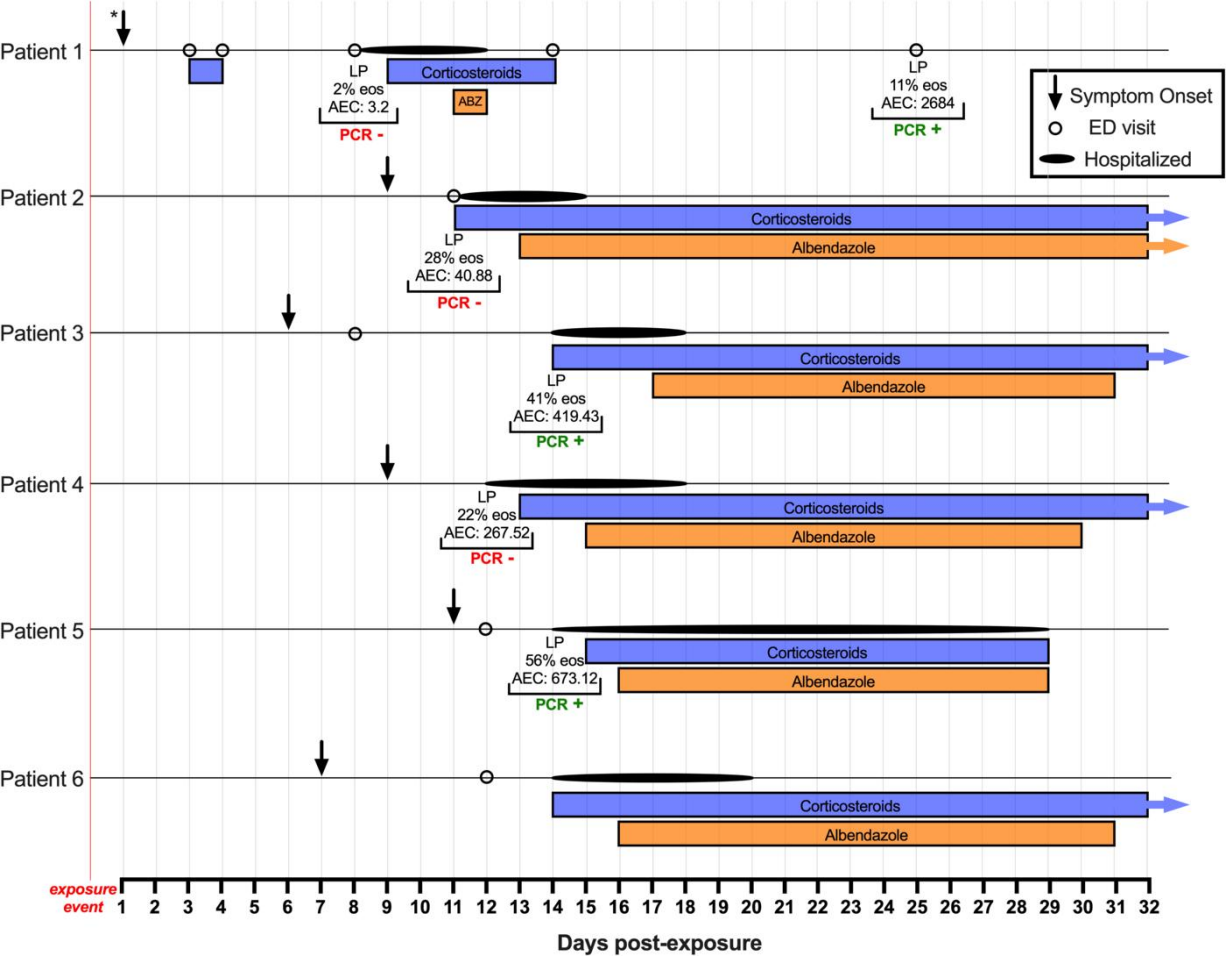
Time course of illness and treatment in Patients 1 through 6, including symptom onset, emergency department visits, duration of hospitalization, eosinophilia, and polymerase chain reaction test results from lumbar punctures. The exposure event is described as a group consumption of a communal kava drink. * Patient 1’s onset of symptoms is noted as the first day post-exposure; however, the exact date for the onset of Patient 1’s symptoms could not be determined. Patients 2, 3, 4, and 6 received high-dose corticosteroids beyond 32 days post-exposure. Patient 2 received albendazole beyond 32 days post-exposure. ABZ = albendazole; AEC = absolute eosinophil count (cell/*μ*L); ED = emergency department; EOS = cerebrospinal fluid eosinophils; LP = lumbar puncture.

The six men of Tongan origin were living on the southeastern side of Hawaiʻi Island, where the majority of NAS cases have been reported in the State of Hawai‘i.^18^ Their exposure to *A. cantonensis* occurred in the early hours of April 1, 2017, when they shared a kava preparation that was later found to contain a dead slug. All the patients were seen at Hilo Benioff Medical Center (formerly Hilo Medical Center). Pertinent medical details, including laboratory findings and the onset and duration of treatment of all six patients, are shown in Table 1. The course of the disease and treatment timeline are shown in Figure 2.

**Table 1:** Disease summary and medical records of patients with neuroangiostrongyliasis

Patient No.	Age (years)	Day of Initiation of Corticosteroids Post-Exposure	Day of Initiation of Albendazole Post-Exposure	Albendazole Regimen	High-Dose Corticosteroid Regimen (days)	Duration of Co-Therapy (days)	Eosinophilia*		PCR Results
							Blood^†^	CSF	
1	36	Second to third day, then ceased; reinitiated on the ninth day^‡^	11th day	400 mg BID for 2 days^§^	48^‖^	2	2–9.3% (AEC: 0.65–2.51)	2% first LP, clear (AEC: 3.2); 11% second LP, clear (AEC: 2,684)	First PCR–, second PCR+
2	46	11th day	13th day	400 mg TID for 23 days	35	23	0.1–15.3% (AEC: 0.01–1.46)	28%, clear (AEC: 40.88)	PCR–
3	30	14th day	17th day	400 mg BID/TID for 14 days	45	14	0.3–13.3% (AEC: 0.03–1.35)	41%, hazy (AEC: 419.43)	PCR+
4	26	13th day	15th day	400 mg TID for 15 days	42	16	0.7–6.5% (AEC: 0–0.66)	22%, hazy (AEC: 267.52)	PCR–
5	52	15th day	16th day	400 mg TID for 14 days	15	14	3.3–26.1% (AEC: 0.29–2.64)	56%, hazy (AEC: 673.12)	PCR+
6	27	14th day	16th day	400 mg TID for 16 days	27	16	4.3–6.7% (AEC: 0.4–0.52)	N/A	N/A

AEC = absolute eosinophil count (cells/*μ*L); BID = two times a day; CSF = cerebrospinal fluid; LP = lumbar puncture; PCR = polymerase chain reaction; TID = three times a day.

* Percentage of eosinophils of total white blood cells.

^†^ Peripheral blood counts are not necessarily from the same day as the lumbar punctures.

^‡^ Administered for his wheezing.

^§^ Patient was unable to receive discharge medication due to a lack of health insurance.

^‖^ Patient compliance is unknown.

### Patient 1.

A 36-year-old man with a known history of gastroesophageal reflux disease, tobacco use, and anxiety, presented to the emergency department (ED) 3 days after drinking kava with body aches, fever, chills, cough, and wheezing. The onset of symptoms occurred 3 to 4 days before presentation. Because of the uncertainty in the timeline based on the patient’s history and his nonspecific symptoms, the incubation period for this patient remains unclear. He was treated with corticosteroids and bronchodilators for his respiratory symptoms and sent home. He returned to the ED the next day (i.e., 4 days after drinking kava) with a persistent cough, dyspnea, and what the ED physician reported as “radiculitis.” An attempt at a lumbar puncture (LP) was unsuccessful. He was treated again with corticosteroids and bronchodilators and sent home with additional corticosteroids. Three days later (now 8 days after drinking kava), he returned to the ED with worsening body aches, left pedal edema, left leg pain, and generalized pruritus. He was hospitalized and underwent a successful LP. His CSF revealed a total WBC count of 160/*µ*L with 2% eosinophils and an absolute eosinophil count (AEC) of 3.2 cells/*µ*L. Real-time polymerase chain reaction testing of his CSF yielded negative results for *A. cantonensis* DNA. The next day, he was treated for presumed NAS with high-dose corticosteroids for 2 days, followed by two doses of albendazole in conjunction with high-dose corticosteroids. He was discharged after 4 days in the hospital and prescribed 4 additional weeks of albendazole and a 14-day course of corticosteroids. However, he could not afford albendazole because of a lack of health insurance. One day after hospital discharge, he was prescribed additional corticosteroids to be taken over 30 days; his adherence to the full course of corticosteroids is not known. Thirteen days after being discharged from the hospital (and now 25 days after drinking kava), he returned to the ED with a persistent severe headache. He underwent a third LP, and his CSF revealed a total WBC count of 24,400/*µ*L with 11% eosinophils (AEC of 2,684 cells/*µ*L). Repeat RTi-PCR testing of his CSF yielded positive results for *A. cantonensis* DNA. Over the subsequent months, the patient returned to the ED several times for headaches. This patient was initially classified as a presumptive case and later reclassified as a confirmed case.

### Patient 2.

A 46-year-old man developed headaches, abdominal pain, bilateral arm pain, and nausea 9 days after drinking kava. Two days later, he presented to the ED and was hospitalized for presumed NAS. An LP revealed a total CSF WBC count of 146/*µ*L with 28% eosinophils (AEC of 40.88 cells/*µ*L). Real-time polymerase chain reaction testing of his CSF yielded negative results for *A. cantonensis* DNA. He was treated with high-dose corticosteroids for 2 days, after which albendazole was added to his regimen. After 5 days of hospitalization, the patient was discharged with albendazole and high-dose corticosteroids to complete a 23-day course of co-therapy. This patient was classified as a presumptive case of NAS.

### Patient 3.

A 30-year-old man developed pain and tenderness in his extremities 6 days after drinking kava. Two days later, he presented to the ED and was treated with analgesics and muscle relaxants for undifferentiated pain. Six days later (now 14 days after drinking kava), he returned to the ED with headache, body aches, and paresthesia and was hospitalized for presumptive NAS. Upon admission, he reported finding the remains of at least one slug at the bottom of the second bucket of kava, which was shared among the six friends. An LP revealed hazy CSF with a WBC count of 1,023/*µ*L and 41% eosinophils (AEC of 419.43 cells/*µ*L). Real-time polymerase chain reaction testing of his CSF yielded positive results for *A. cantonensis* DNA. He was treated with a high-dose corticosteroid for 3 days, after which albendazole–corticosteroid co-therapy was initiated. He was discharged on the fifth day of hospitalization with albendazole and high-dose corticosteroids and instructed to complete a 14-day course of co-therapy. This patient was classified as a confirmed case of NAS.

### Patient 4.

A 26-year-old man developed a headache, body aches, cough, and insomnia 9 days after drinking kava. Three days later, he was hospitalized for possible meningitis after being associated with the communal event. An LP revealed hazy CSF with a WBC count of 1,216/*µ*L and 22% eosinophils (AEC of 267.52 cells/*µ*L). Real-time polymerase chain reaction testing of his CSF yielded negative results for *A. cantonensis* DNA. On the second day of hospitalization, his headache worsened, and he developed photophobia and phonophobia. He was administered a high-dose corticosteroid for 2 days, followed by albendazole–corticosteroid co-therapy. He was discharged on the seventh day of hospitalization with instructions to complete a 16-day course of albendazole corticosteroid co-therapy, followed by high-dose corticosteroid monotherapy for 5 weeks. This patient was classified as a presumptive case of NAS.

### Patient 5.

A 52-year-old man developed left upper quadrant pain and vomiting 11 days after drinking kava. One day later, he presented to the ED, where he was diagnosed with hyponatremia and discharged home. One day later, he returned to the ED with a severe headache, neck stiffness, persistent emesis, abdominal pain, and pain in the right leg. He was hospitalized, and an LP revealed hazy CSF with a WBC count of 1,202/*µ*L and 56% eosinophils (AEC of 673.12 cells/*µ*L). Real-time polymerase chain reaction testing of his CSF yielded positive results for *A. cantonensis* DNA. On the second day of hospitalization, a high-dose corticosteroid was initiated, and the patient underwent a therapeutic LP to relieve his severe headache; the CSF was not analyzed. After 24 hours of high-dose corticosteroids, albendazole–corticosteroid co-therapy was initiated for 14 days. This patient was hospitalized for 16 days and received a course of co-therapy as an inpatient. This patient was classified as a confirmed case of NAS.

### Patient 6.

A 27-year-old man with a history of asthma developed left-sided body pain and paresthesia that started 7 days after drinking kava. Five days later, he presented to the ED and was sent home with analgesics and bronchodilators. One day later, he was hospitalized for a headache, body aches, chest pain, shoulder pain, and persistent paresthesia. An LP was unsuccessful. He received high-dose corticosteroids for 2 days, followed by co-therapy with albendazole and corticosteroids. He was discharged on the seventh day of hospitalization with albendazole and high-dose corticosteroids (for a total of 16 days of co-therapy). This patient was classified as a presumptive case of NAS.

#### Epidemiological investigation.

On April 15, 2017, two field investigators from the Hawaiʻi Department of Health’s (HDOH) district office visited the site of kava consumption. They discovered that the kava was prepared using tap water from the municipal supply. The dried kava root, sourced from Tonga, was placed into two sachets, each of which was soaked in a 5-gallon plastic bucket of water. These buckets were left outdoors, uncovered, on the ground during the event. No details were recorded about how the sachets were cleaned and dried.

The men reported transferring portions from the first bucket into the serving bowl. After consuming the preparation from the first bucket, they began drinking from the second bucket. As they poured the last portion from the second bucket into the serving bowl, they noticed a slug at the bottom. The slug was discarded and not available for testing.

Numerous slugs, tentatively identified as Cuban slugs (*Veronicella cubensis*) and semi-slugs (*Parmarion martensi*), were found in the area surrounding the water hose and spigot used to prepare the kava, as well as on the adjacent lawn. Residents of the property were provided with information on how to control gastropods in and around the dwelling and yard. The gastropod species identified on the property have been previously reported to be heavily infected with *A. cantonensis.*^22^^,^^23^ Additionally, rats in this area of the Big Island have a high prevalence of infection.^24^

## DISCUSSION

In the present study, a cluster of six NAS cases in an enzootic region of Hawaiʻi Island with a high prevalence of infected definitive and intermediate hosts is described. All patients were infected after consuming kava that was found to be contaminated with at least one slug, which was presumably infected with *A. cantonensis*. To the best of the authors’ knowledge, the present report is the first documentation of a group of patients treated with albendazole and corticosteroids within 15 days of infection. Importantly, this early treatment was effective and well-tolerated, supporting the belief that initiating albendazole and corticosteroid treatment for NAS as early as possible, ideally within 15–21 days of exposure, is crucial.^25^^,^^26^

This is also the first report of a cluster of NAS cases associated with drinking contaminated water, supporting a waterborne transmission pathway in the epidemiology of this parasitic infection. The consumption of non-potable freshwater contaminated with L3 larvae of *A. cantonensis* released from terrestrial mollusks has been proposed as a potential route of infection.^27^ This method of transmission is supported by a previously documented common-source outbreak of EOM linked to consumption of vegetable juice, although the source of contamination was not confirmed.^28^ Recently, experimental work by Howe et al.^29^ revealed that L3 larvae released from deceased gastropods can remain viable in freshwater for prolonged periods. In the present report, the property solely relied on the city’s municipal water supply, thereby excluding catchment water as a potential source of contamination.

The epidemiological investigation revealed numerous slugs in the area around the event. A slug is suspected to have crawled into the second bucket of kava, which remained unprotected throughout the night and early morning of the gathering. The gastropod went unnoticed and drowned, releasing *A. cantonensis* L3 larvae into the kava preparation. Although the gastropod is suspected to have crawled into the second kava bucket, it cannot be ruled out that the slug came from the water hose, spigot, or the sachet bags used to strain the kava. Additionally, confirmation of the source was not possible because the slug and the contaminated kava from the incident were unavailable for testing.

The incubation period for NAS has been reported to range from 1 day to several weeks.^3^^,^^12^ In the present report, the average incubation period (Patients 2–6) was 8.4 days (SD ± 1.95). The chronology of Patient 1’s symptoms was not entirely clear from the available records, so he was excluded from the calculation of the incubation period. Although short incubation periods can occur, it is more likely that Patient 1’s initial symptoms of wheezing, cough, and dyspnea were related to a pre-existing condition or illness. Nonetheless, it remains possible that he experienced prodromal symptoms on the day of the event, provoked by L3 larvae migrating from the intestine throughout the body and into the CNS.^25^^,^^30^ Prodromal symptoms accompanying the early stages of *A. cantonensis* infection are often diverse and nonspecific. Unless clinicians possess a high index of suspicion (or there is a known ingestion of a snail or slug), such symptoms are rarely attributed to *A. cantonensis* infection. This was evident in Patient 1, who was the index case and the first among the cluster of six to present for evaluation. His symptoms and vague history made it difficult to determine his incubation period with any certainty. However, it can reasonably be assumed that his incubation period was likely between 1 and 4 days because his second presentation to the ED (day 4 post-exposure) revealed more specific CNS symptoms with radiculitis, along with an unsuccessful LP attempt, indicating a higher level of suspicion for NAS.

Headache is the most common presenting NAS symptom. The headaches are usually acute in onset and described as either throbbing in quality or as a steady pain lasting minutes to hours in the temporal, frontal, or occipital regions.^13^^,^^31^^,^^32^ The severity of headache may correlate with cellularity, elevated CSF protein levels, and elevated intracranial pressure.^31^ Headache is often accompanied by neck stiffness and signs of meningeal irritation, as well as phonophobia and photophobia. All six patients in the present series experienced headaches at some point in their illness.

The diagnosis of NAS is often delayed because the disease is rare and its clinical manifestations can be variable. Additionally, it is often challenging to determine the precise time and date of infection in most cases of NAS. Moreover, many healthcare providers—especially in regions where *A. cantonensis* has only recently emerged—lack familiarity with the disease.

At the time of the incident, the patients described in this report were living on Hawai‘i Island, where clinicians at the Benioff Hilo Medical Center were familiar with the clinical features of NAS. Once the connection between suggestive symptoms and possible exposure was established, the six men were admitted, a presumptive diagnosis was made, and treatment of NAS was promptly initiated.

Although the definitive diagnosis for NAS is recovery of the parasite from the CSF, this rarely occurs.^13^ Therefore, when available, polymerase chain reaction (PCR) testing is used to detect *A. cantonensis* DNA in CSF to confirm the diagnosis.^21^ Eosinophilic pleocytosis has been the hallmark of EOM, including EOM caused by *A. cantonensis.*^19^^,^^20^ However, in a recent report by Hiraoka et al., (2020)^33^ NAS is recommended for consideration in patients with compatible signs and symptoms, regardless of the degree of CSF eosinophilia, particularly in those with potential exposure. The utility of this recommendation is supported by Patient 1, whose initial CSF contained 2% eosinophils and whose PCR test results were negative 7 days post-exposure. The CSF from his subsequent LP (24 days post-exposure) revealed 11% eosinophils, and the patient’s second PCR test yielded positive results for *A. cantonensis* DNA.

At the time of this outbreak, the widely accepted definition of EOM was the presence of 10 or more eosinophils per microliter of CSF, or eosinophils accounting for more than 10% of WBCs. However, the presence of any eosinophils in the CSF is indicative of pathology. If the EOM criteria of 10% eosinophilia are used as a threshold for testing for *A. cantonensis*, PCR testing might be delayed and could potentially result in a delay in treatment as well. Thus, clinicians should not rely on a threshold of 10% eosinophilia before considering the diagnosis of NAS.

In this report, five of six patients underwent successful LPs between 7 and 24 days post-exposure, with eosinophilic pleocytosis ranging from 2% to 56%. Three of the five patients with successful LPs had positive RTi-PCR test results for *A. cantonensis* (Patients 1, 3, and 5). In comparison, the remaining two (Patients 2 and 4) were presumptively diagnosed on the basis of their CSF eosinophilia (41% and 56%, respectively), compatible signs and symptoms, and participation in the common exposure event. The negative RTi-PCR test result may have been due to sub-detection threshold levels of* A. cantonensis* DNA at the time of the LP. Of note, negative RTi-PCR test results from CSF specimens were obtained 8, 11, and 12 days after infection, and the CSF specimens with positive RTi-PCR test results were obtained 14, 14, and 25 days after infection. The newly developed AcanR3990 PCR assay may help overcome this limitation because of its increased sensitivity.^34^ The HDOH recently adopted the AcanR3900 PCR test and will test CSF samples from symptomatic patients with relevant exposure histories, regardless of the CSF eosinophil count.^35^ Although tools such as computed tomography (CT)^36^ and magnetic resonance imaging (MRI)^37^^,^^38^ scans of the brain have been reported to be useful for the diagnosis of NAS in some cases, there are no pathognomonic findings, and brain imaging alone neither confirms nor rules out NAS. Three of the patients (Patients 1, 4, and 6) in the present study had brain CTs, and one (Patient 1) had a brain MRI; however, none of the scans provided any conclusive information.

Albendazole is considered the anthelminthic of choice for many parasitic infections of the CNS because of its broad spectrum of anthelmintic activity and ability to cross the blood–brain barrier.^39^^,^^40^ The use of anthelminthics for the treatment of NAS has been debated because of the concern that administering albendazole would kill the larvae in the CNS and trigger an intense inflammatory response. A recent comprehensive literature review of clinical reports and animal studies revealed that in nine of nine published reports on 743 patients and 479 animals with confirmed NAS (diagnosed via PCR testing, immunodetection, or larval recovery), albendazole monotherapy was effective in reducing symptoms and resulted in no serious adverse events.^41^ Although this review revealed no evidence of adverse events with albendazole monotherapy, high-dose corticosteroid co-administration is still recommended because of its potential to provide symptomatic relief and to allay concerns about potential inflammation caused by dying larvae.

The authors of the present study believe that early administration of albendazole is essential for reducing long-term morbidity and sequelae. Animal studies have revealed that albendazole is most effective at reducing the number of L5-stage larvae when administered within 15 days of infection.^26^ Thus, the early administration of albendazole may stop the growth, development, and migration of the parasite in the CNS.

High-dose corticosteroids have been found to reduce the duration of headaches in patients with NAS in Thailand.^42^ Patients in the present report received several different corticosteroids, including dexamethasone, methylprednisolone, prednisolone, and prednisone, administered as monotherapy for 24 to 72 hours before the addition of albendazole. This approach was designed to mitigate potential CNS inflammation that might develop after the introduction of the larvicidal anthelminthic drug. Although there is no concrete evidence that such an approach is necessary,^41^ the authors support albendazole–corticosteroid co-therapy initiation as soon as possible after a presumptive diagnosis of NAS.^43^ It should also be noted that because corticosteroids suppress eosinophilia, measurements of eosinophils obtained after the administration of high-dose corticosteroids may be falsely low.

With respect to treatment duration, the patients reported here received at least a 2-week course of high-dose corticosteroids (although the compliance of Patient 1 is unknown), and five of the six patients received at least a 2-week course of albendazole–corticosteroid co-therapy. High-dose corticosteroid treatment was initiated 7 to 15 days (mean 11.9 days) post-exposure. Similarly, albendazole treatment (15 mg/kg/day^41^^,^^44^) was initiated 10 to 16 days (mean 13.5 days) post-exposure (Figure 2). Patient 1 only received two doses of albendazole (because of the high cost of albendazole^45^ and the patient’s lack of insurance), and he experienced recurrent symptoms, including severe headache on two occasions (day 24 and day 45 post-exposure). All six patients tolerated co-therapy with albendazole and corticosteroids, with no adverse side effects observed. Long-term follow-up was not available, but it was anecdotally reported that all patients eventually returned to their jobs in the construction industry.

## CONCLUSION

Neuroangiostrongyliasis is a preventable disease, and public health campaigns in endemic areas should educate residents and visitors on how to reduce the risk of infection. Additionally, these findings underscore the need for community education on safe kava preparation (or any other consumable preparations made outdoors) and gastropod control to reduce NAS in endemic areas. Detailed information on prevention can be found on the State of Hawaiʻi’s Department of Health, Disease Prevention Division website.^46^
